# A New Perspective on Low-Cost MEMS-Based AHRS Determination

**DOI:** 10.3390/s21041383

**Published:** 2021-02-16

**Authors:** Neda Navidi, Rene Landry

**Affiliations:** LASSENA Laboratory, Department of Electrical Engineering, Ecole de Technologie Superieure, Montreal, QC H3C 1K3, Canada; renejr.landry@etsmtl.ca

**Keywords:** inertial navigation system, adaptive Kalman filter, inertial measurement unit

## Abstract

Attitude and heading reference system (AHRS) is the term used to describe a rigid body’s angular orientation in three-dimensional space. This paper describes an AHRS determination and control system developed for navigation systems by integrating gyroscopes, accelerometers, and magnetometers signals from low-cost MEMS-based sensors in a complementary adaptive Kalman filter. AHRS estimation based on the iterative Kalman filtering process is required to be initialized first. A new method for AHRS initialization is proposed to improve the accuracy of the initial attitude estimates. Attitude estimates derived from the initialization and iterative adaptive filtering processes are compared with the orientation obtained from a high-end reference system. The improvement in the accuracy of the initial orientation as significant as 45% is obtained from the proposed method as compared with other selected techniques. Additionally, the computational process is reduced by 96%.

## 1. Introduction

The determination of Attitude and Heading Reference System (AHRS) involves several fields like navigation, control, motion tracking [1,2,3], personal navigation [4,5], robotics [6], and virtual reality systems [7]. Several AHRS determination and control technologies in use need an external source to obtain orientation information [1]. Interference and shadowing are the main problems associated with these technologies. Compared with other technologies, the inertial system computes the attitude using self-contained sensors that only respond to inertial forces.

In inertial systems, the attitude is derived from the integration of rate gyroscope data in an inertial system. Rate gyroscopes are prone to bias and random drifts and this leads to unbounded attitude errors. Thus, successful implementation of an inertial system requires very expensive sensors that have exceptional long-term bias stability [8]. In the last decade, the rapid development of Micro-Electro-Mechanical System (MEMS) inertial sensors in precision, accuracy, size, weight, and cost make it ideal for developing a small-scale and low-cost AHRS determination and control system. However, inexpensive MEMS gyroscopes are low-performance, and using these gyroscopes may result in unbounded attitude errors. An AHRS determination and control system can be successfully implemented using such gyroscopes if there is a means for aiding the gyroscopes or resetting the attitude errors periodically [8].

One of the most successful applications for aiding the low-cost inertial sensors is the use of the Global Positioning System (GPS). GPS data are precise, and the errors are independent of time. This makes it ideal to calibrate the drift errors of low-cost gyroscopes. However, GPS has well-known limitations due to signal attenuation in indoor applications, so other technologies like the earth’s magnetic and gravitational sensing [9] can help improving navigation systems.

Research is currently being undertaken in many laboratories for navigational tracking using low-cost MEMS-based inertial sensors using advanced signal processing techniques to improve the performance of such sensors [10]. The method to integrate the data from gyroscopes and the aiding devices is Kalman filtering, in which the sources of information are weighted appropriately with knowledge about the signal characteristics.

Accelerometers measure the sum of translational acceleration and earth gravity. However, the gravity is more significant than the translational acceleration in most situations [11,12,13,14] presented a method of fusing MEMS-based low-cost gyroscopes and accelerometers in a Kalman filter for orientation estimation. In addition, magnetometers can be used to correct the drifts in heading estimate from gyroscopes due to their sensitivity to the earth’s magnetic field [15].

References [1,16,17] also presented the development of different kinds of Kalman filters to prevent gyroscope drift and the gyroscope data are integrated to monitor the variation of orientation between successive measurements. When a movement increases, accelerometers’ discrimination becomes difficult, as they are sensitive to both gravity and translational acceleration. To separate these components, [6,18] suggested testing the acceleration magnitude for significant deviation from gravity. Magnetometers are subject to magnetic disturbances and this causes large errors in the heading. [1,3,4] proposed the use of various magnetic error models in the filter to detect the ambulatory magnetic disturbances and provide on-line calibrations.

This paper proposes a new method for AHRS determination and control system using MEMS-based low-cost and small-scale sensors for tracking and monitoring moving object-related applications. The main contributions of this paper are:

(1) AHRS initialization process is applied under stationary conditions to determine the initial orientation. We developed a new method for AHRS initialization, named Hybrid Method to increase accuracy and precision.
(2) An adaptive Extended Kalman filter (AEKF)-based iterative process is applied to perform the real-time estimation of attitude when the sensor is in motion. While gyroscope measurements are integrated to yield the attitude changes between successive body movements to maintain the high-frequency output of attitude, accelerometers and magnetometers provide a low frequency and noisy but drift-free calibration of attitude. The developed AEKF is adaptive to the effects of body acceleration and magnetic interferences, named the real-time calibration process.

## 2. Materials and Methods

In body frame (*B*), a sensor unit rotates and translates with respect to a navigation coordinate frame (*N*). The attitude of the sensor can be analytically represented by a direction cosine matrix $C_{B}^{N}$ that transforms an arbitrary 3 × 1 vector A from its coordinate frame *B* (projection $A^{B}$) to the *N* frame (projection $A^{N}$), presented by Wahba’s problem [11] as:

(1) $$A^{N}=C_{B}^{N}A^{B}$$

when the body is in motion, its attitude relative to the reference *N* frame can be represented by a time-varying function of $C_{B}^{N}\left(t\right)$. AHRS initialization is the process to determine the initial value of $C_{B0}^{N}$.

In this study, we consider a two-step process, a coarse alignment followed by fine alignment for AHRS initialization. In the following section, we introduce geomagnetic matching, Compass Heading, and our new method to achieve coarse alignment. In the end, fine alignment is designed to iteratively estimate the residual error in the heading estimation.

### 2.1. Geomagnetic Matching Technique in Coarse Alignment

Geomagnetic matching, one of the techniques often used in determine attitude [8,16], is based on solving Equation (1). While MEMS-based low-cost gyroscopes fail to sense the earth’s rotation, the earth’s magnetic and gravitational acceleration vectors are independent and thus can be used in the AHRS initialization process.

Accelerometers in the static state can measure the components of gravity in *B* frame ($g^{B}$), and its *N* frame projection ($g^{N}$) is given by the standard gravity model [19]. Additionally, magnetometers sense the earth’s magnetic vector in *B* frame ($m^{B}$) and its *N* frame projection can be obtained from international geomagnetic reference field models [20]. $C_{B0}^{N}$ can be obtained with $g^{N}=C_{B0}^{N}g^{B}$, $m^{N}=C_{B0}^{N}m^{B}$, and $h^{N}=C_{B0}^{N}h^{B},$ where, $h^{N}=g^{N}\times m^{N}$, and $h^{B}=g^{B}\times m^{B}$, × is the cross-product between gravity and the earth’s magnetic vector [8].

### 2.2. Compass Heading Technique in Coarse Alignment

Compass heading, another technique used to initialize the heading. The earth’s magnetic field has a component in the local horizontal plane that is always pointing toward the magnetic north. If a triaxial magnetometer is placed in the horizontal plane, the heading can be calculated using the horizontal components of the earth’s magnetic field. When the magnetometer is tilted, the tilt angles should be first compensated for before calculating the heading [21].

The tilt angles represented in by [21] give us roll (ϕ) and pitch (θ), calculated by: $\phi =tan^{-1}\left(y/z\right)$, and $\theta =tan^{-1}\left(x/\sqrt{y^{2}+z^{2}}\right)$, where x,y,z are the components of gravity in B coordinate frame. The magnetometers data can be calculated in the horizontal plane by Xh=xcosθ+ysinϕsinθ+zcosϕsinθ and Yh=ycosϕ−zsinϕ, where x,y,z are readings of the triaxial magnetometer. In the end, heading angle ψ can be determined by $\psi =tan^{-1}$. Using the equivalence relation between the direction cosine matrix and the Euler angle parameters, the attitude matrix $C_{B0}^{N}$ can be initialized in terms of ψ,ϕ,θ [21]:

(2) $$C_{B0}^{N}=\left[\begin{array}{ccc} C_{11} & C_{12} & C_{13}\\ C_{21} & C_{22} & C_{23}\\ C_{31} & C_{32} & C_{33} \end{array}\right]\;=\left[\begin{array}{ccc} cos\theta cos\psi & -cos\phi sin\psi +sin\phi sin\theta cos\psi & sin\phi sin\psi +cos\phi sin\theta cos\psi \\ cos\theta sin\psi & cos\phi cos\psi +sin\phi sin\theta sin\psi & -sin\phi cos\psi +cos\phi sin\theta sin\psi \\ -sin\theta & sin\phi cos\theta & cos\phi cos\theta \end{array}\right]$$

### 2.3. Hybrid Method in Coarse Alignment

The new method for AHRS initialization proposed by this is study is developed based on the geomagnetic matching technique defined in Equation (1). As ${\left(C_{B}^{N}\right)}^{-1}={\left(C_{B}^{N}\right)}^{T}=C_{N}^{B}$, Equation (1) can be rearranged to $A^{B}=C_{N}^{B}A^{N}$, and attitude matrix $C_{N}^{B}$ is represented in Equation (2).

If an arbitrary vector V is specified as the unit vectors along the *X*, *Y*, and *Z* axes of the coordinate frame *N*, the *B* frame projections of the unit vectors along *N* frame *X*, *Y*, and *Z* axes are expressed in terms of the elements of $C_{B}^{N}$ as:

(3) $$V_{XN}^{B}=\left[\begin{array}{c} cos\theta cos\psi \\ -cos\phi sin\psi +sin\phi sin\theta cos\psi \\ sin\phi sin\psi +cos\phi sin\theta cos\psi \end{array}\right],V_{YN}^{B}=\left[\begin{array}{c} cos\theta sin\psi \\ cos\phi cos\psi +sin\phi sin\theta sin\psi \\ -sin\phi cos\psi +cos\phi sin\theta sin\psi \end{array}\right],V_{ZN}^{B}=\left[\begin{array}{c} -sin\theta \\ sin\phi cos\theta \\ cos\phi cos\theta \end{array}\right]$$

**Figure 1:**
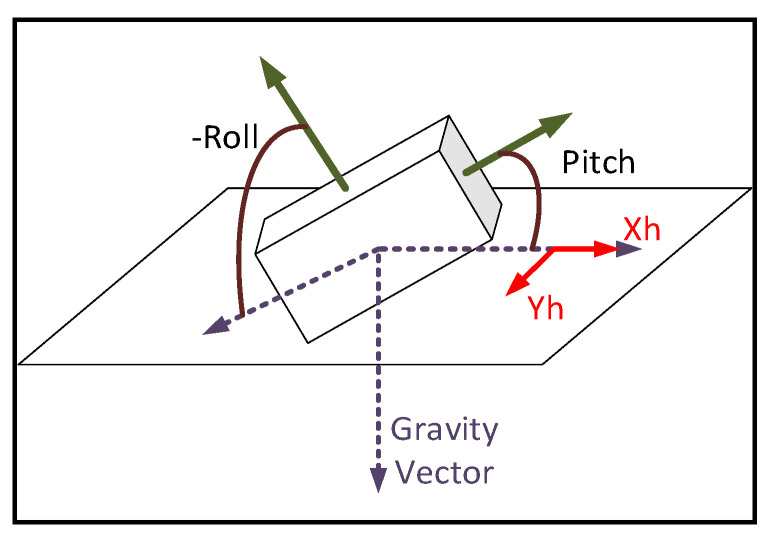
The gravity and magnetic vectors shown in N coordinate frame.

So, by $C_{B}^{N}=\left[\begin{array}{c} {\left(V_{XN}^{B}\right)}^{T}\\ {\left(V_{YN}^{B}\right)}^{T}\\ {\left(V_{ZN}^{B}\right)}^{T} \end{array}\right],$ can find the gravity vector g is aligned to the *Z* axis of the *N* frame and the earth’s magnetic vector m lies in the *X*–*Z* plane depicted in Figure 1.

The *X*, *Y*, and *Z* axes are mutually orthogonal; thus, the earth’ magnetic vector m is perpendicular to the *Y*-axis. Vectors along the *Y*-axis can therefore be computed from vector cross product between g and m. Realizing that $g^{B}$ and $m^{B}$ are the readings of the accelerometer and magnetometer when the body remains static during the initialization, the unit vector along the N frame *Y*-axis projected on B frame axes can be calculated by $V_{g}^{B}\times V_{m}^{B},$ where, $V_{g}^{B}$ is the B frame projection of the unit gravity vector and $u_{m}^{B}$ is the unit earth’s magnetic vector. So, $V_{XN}^{B}$ can be computed by $V_{YN}^{B}\times V_{g}^{B}$. So $C_{B0}^{N}$ can be initialized by $\left[\begin{array}{c} {\left(V_{g}^{B}\times V_{m}^{B}\times V_{g}^{B}\right)}^{T}\\ {\left(V_{g}^{B}\times V_{m}^{B}\right)}^{T}\\ {\left(V_{g}^{B}\right)}^{T} \end{array}\right]$.

## 3. Fine Alignment Process

Fine alignment process due to estimate the iterative orientation is implemented using Kalman filtering technique. The adaptive extended version of the Kalman filter (EKF) is developed in this study due to the high nonlinearity of the system. The state vector is defined to contain attitude and inertial sensor error, presented by $x={\left[\begin{array}{cccc} q_{e} & b_{\omega }^{B} & b_{a}^{B} & d_{m}^{B} \end{array}\right]}^{T}$, where, $q_{e}$ is quaternion vector of attitude errors, $b_{\omega }^{B}$ is gyroscopes random errors, $b_{a}^{B}$ is acceleration errors, and $d_{m}^{B}$ is magnetic disturbances.

The system model is a time-varying function of angular velocity and accelerations. When the initial value of attitude is obtained, 1st order approximation can be safely applied for remaining residual attitude errors. The attitude errors are defined by first rotating the body by an amount equal to the current attitude estimate [22]. By considering $q=\hat{q}⨂q_{e},\;$ where, q is the attitude quaternion, the combination of a vector component q and a scalar component $q_{0}$, and ⨂ indicates quaternion multiplication [23]; $\hat{q}$ is the estimated attitude; $q_{e}$ represents the small error in attitude, in view of quaternion definition, it is approximated as $q_{e}=\left(q_{e},1\right)$.

For the high-frequency changes of attitude, we should consider $\overset{´}{q}=0.5q⨂W_{I\leftarrow B}^{B}-0.5W_{I\leftarrow N}^{N}⨂q$ where, $W_{I\leftarrow B}^{B}=\left(\omega_{I\leftarrow B}^{B},0\right),W_{I\leftarrow N}^{N}=\left(\omega_{I\leftarrow N}^{N},0\right).\;W_{I\leftarrow B}^{B}$ and $\omega_{I\leftarrow N}^{N}$ are the quaternion form of the angular velocity of the *B* frame and *N* frame, respectively; relative to the inertial (*I*) frame.

$\omega_{I\leftarrow N}^{N}$ is dominated by the earth’s rotation rate, the translational movement of the body, and the earth’s local curvature. In the AHRS determination, $\omega_{I\leftarrow N}^{N}$ is negligible compared to the errors of low-cost gyroscopes; so, $\overset{´}{q}=0.5q⨂W_{I\leftarrow B}^{B}-0.5W_{I\leftarrow N}^{N}⨂q$ can be written as $0.5q⨂W_{I\leftarrow B}^{B}$. Additionally, the propagation equation of the errors is derived after neglecting the 2nd order terms of $q_{e}$, can be simplified as ${\overset{´}{q}}_{e}=-0.5\left(\omega_{I\leftarrow B}^{B}\times \right)q_{e}$, where ($\omega_{I\leftarrow B}^{B}\times )$ is the skew-symmetric matrix of vector $\omega_{I\leftarrow B}^{B}$.

A measurement model is the analytical representation of the actual input to a Kalman filter.

In this study, we consider different inputs of accelerometer, gyroscope, and magnetometer data as the input to the Kalman filter. The general vector notation A is used to represent the true gravity or magnetic vector [8]. $\delta A^{N}=-2\left(A^{N}\times \right)q_{e}$, that $A^{N}$ and $\delta A^{N}$ represent vector A is projected on N frame, and the error of $A^{N}$, respectively. According to [8], we can consider $\delta A^{N}=A^{N}-{\hat{C}}_{B}^{N}A^{B},$ where, ${\hat{C}}_{B}^{N}$ and $A^{B}$ are the estimated attitude and the true gravity or magnetic vectors, respectively.

Ref. [8] presents that the general linear measurement model can be considered as $\delta {\tilde{A}}^{N}=-2{\hat{C}}_{B}^{N}\left({\tilde{A}}^{B}\times \right){\left({\hat{C}}_{B}^{N}\right)}^{T}q_{e}-{\hat{C}}_{B}^{N}b^{B}$, that can be simplified to $\delta {\tilde{A}}^{N}=A^{N}-{\hat{C}}_{B}^{N}{\tilde{A}}^{B},$ applying the accelerometer, gyroscope and magnetometer raw measurements. So, the measurement models are defined as:

(4) $$\delta {\tilde{A}}_{a}^{N}=-2{\hat{C}}_{B}^{N}\left({\tilde{A}}_{a}^{B}\times \right){\left({\hat{C}}_{B}^{N}\right)}^{T}q_{e}-{\hat{C}}_{B}^{N}b_{a}^{B}\;\delta {\tilde{A}}_{m}^{N}=-2{\hat{C}}_{B}^{N}\left({\tilde{A}}_{m}^{B}\times \right){\left({\hat{C}}_{B}^{N}\right)}^{T}q_{e}-{\hat{C}}_{B}^{N}d_{m}^{B}\;\delta {\tilde{A}}_{g}^{N}=-2{\hat{C}}_{B}^{N}\left({\tilde{A}}_{g}^{B}\times \right){\left({\hat{C}}_{B}^{N}\right)}^{T}q_{e}-{\hat{C}}_{B}^{N}d_{g}^{B}$$

where ${\tilde{A}}_{a}^{N}$, ${\tilde{A}}_{m}^{N}$ and ${\tilde{A}}_{g}^{N}$ are the data of the triaxial accelerometer, magnetometer, and gyroscope, $b_{a}^{B}$, $d_{m}^{B}$ and $d_{g}^{B}$ are the acceleration errors and magnetic disturbance, and gravity errors.

**Figure 2:**
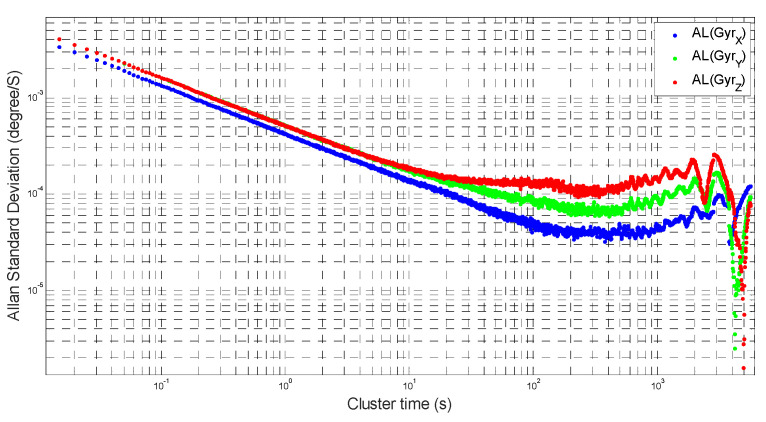
Plot of Allan standard deviation σ(τ) versus cluster times τ.

Regarding the sensor model, this study investigated three stochastic models that are developed to account for the gyroscope random drifts, body acceleration, and magnetic disturbances. Employing Allan variance to analyze the stochastic errors of the low-cost gyroscopes used in this study [24,25], a log–log plot of Allan standard deviation σ(τ) versus cluster times τ is shown in Figure 2.

The results indicate that the dominant noise type appears in the σ(τ)/τ plot with slope of −1/2, which represents the random walk noise term. The gyroscope stochastic error is thus approximated as a random walk process, presented by ${\overset{´}{b}}_{\omega }^{B}=n_{\omega }^{B},\;$ where, $b_{\omega }^{B}$ is a vector representing the stochastic errors of a triaxial gyroscope; $n_{\omega }^{B}$ is a vector of Gaussian white noise with the noise density $\sigma_{\omega }$. The acceleration errors caused by body motion are modeled by a 1st-order Markov random process [3], presented by ${\overset{´}{b}}_{a}^{B}\tau_{a}b_{a}^{B}+n_{a}^{B}$, where $b_{a}^{B}$ is a vector representing the triaxial acceleration errors; $\tau_{a}$ is a diagonal matrix, of which the three diagonal entries are the time constants for the acceleration errors along each axis of the sensor device; $n_{a}^{B}$ is a vector of Gaussian white noise whose density $\sigma_{a}$.

To extract the stochastic noises in IMU, we put the sensor in static mode for about 4 h. When the experiment is performed in an environment where there are no significant external magnetic fields, the magnetic disturbances can be estimated as a 1st-order Gauss–Markov process, ${\overset{´}{d}}_{m}^{B}\tau_{m}d_{m}^{B}+n_{m}^{B},$ where $d_{m}^{B}$ is a random magnetic disturbances vector; $\tau_{m}$ is a diagonal matrix, and its three diagonal elements represent the time constants for the magnetic disturbances along each axis; $n_{m}^{B}$ is Gaussian white noise vector with density $\sigma_{m}$.

## 4. Experimental Results and Discussion

The experiments evaluate the AHRS initialization by different techniques and investigate the accuracy and stability of the AHRS determination and control system in different scenarios. We use MEMS-based inertial sensor unit nIMU, which contains triaxial gyroscope, accelerometer, and magnetometer. Additionally, we utilize an accurate optical motion capturing system Vicon as a true reference. 

### 4.1. AHRS Initialization

AHRS initialization consists of coarse alignment and fine alignment. Coarse alignment approximately obtains the initial orientation. As the heading results derived from coarse alignment may be significantly affected by magnetic disturbances, fine alignment is employed to compensate for the heading errors. So, the EKF-based attitude estimation process is used for the initialization under stationary conditions.

The testing unit is placed on the top of a turning table in eight different setups and the data are recorded at 100 Hz for a duration of 10 s. The three-coarse alignment process using three different techniques is evaluated by comparing the Euler angle parameters of the attitude. Three methods present the same accuracy and precision in estimating heading (ψ) orientation shown in Table 1. Regarding the accuracy, three methods almost identically show significant errors compared with the references. The errors have a mean value of 178.1° and vary between 161.9° and 188.5°. The reason causing the heading errors is the existence of magnetic disturbances.

In this table, pitch (θ) and roll (ϕ) angles, when compared with the approach based on the compass heading or hybrid method techniques, the geomagnetic matching presents significant errors in the pitch and roll angles among some scenarios. The maximum differences between compass heading and the references are 1.5° and 0.8°; whereas the differences are 7.0° and 5.6°, in pitch and roll, respectively, for geomagnetic matching. Regarding the pitch angles, although 50% of scenarios have an estimation error less than 1.0° using the geomagnetic matching method, some trails show significant errors bigger than 6.0°.

The reason for significant errors in estimating pitch and roll attitude using the geomagnetic matching technique is that the gravity measurements are exclusively used to compute the pitch and roll angles in the approaches based on compass heading and hybrid method techniques. Since the accelerometer is stationary during the initialization process, it only measures gravity and thus provides a good estimation of pitch and roll angles.

The adaptive EKF used for fine alignment is applied at the end of the coarse alignment. It is operated under static conditions for a duration of 10 s. The result of the coarse alignment is used to initialize the system.

**Figure 3:**
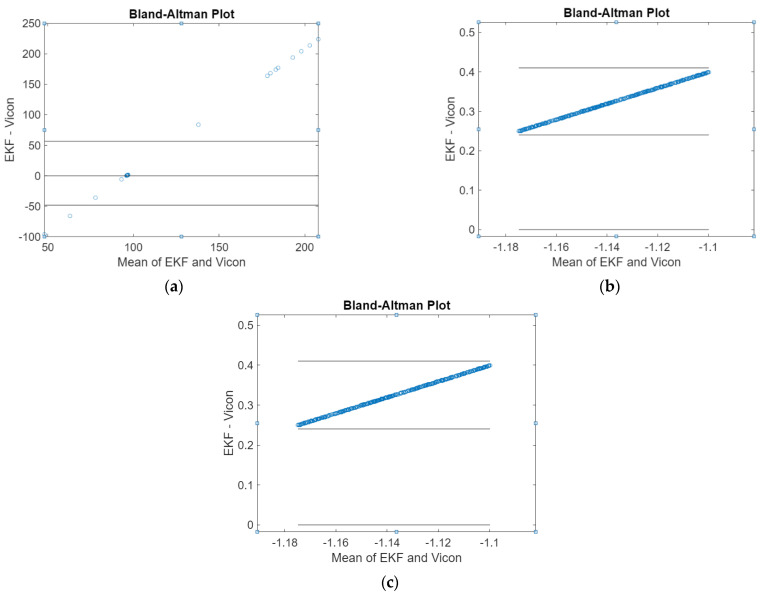
Profile of Euler angles during the fine alignment process (scenario no. 1): (**a**) Heading, (**b**) Pitch, and (**c**) Roll.

**Figure 4:**
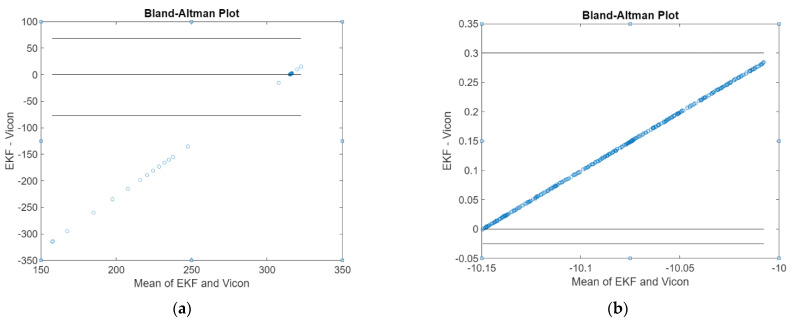

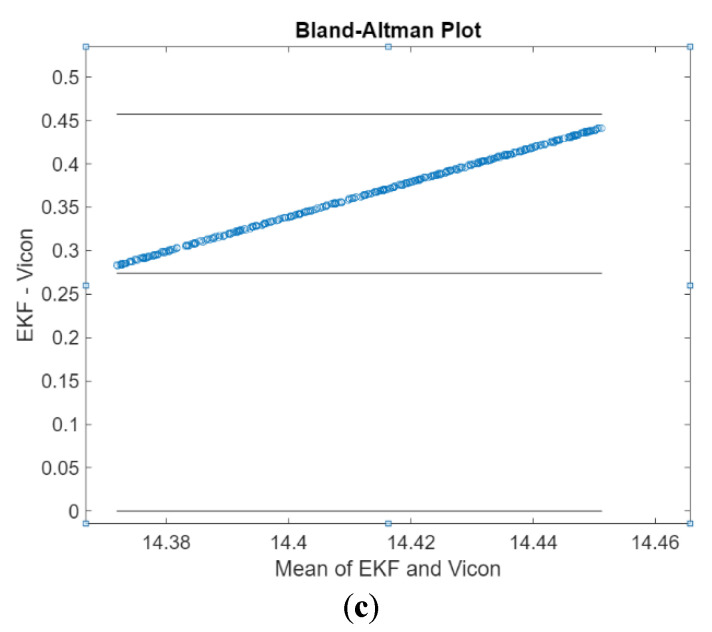
Profile of Euler angles during the fine alignment process (scenario no. 6): (**a**) Heading, (**b**) Pitch, and (**c**) Roll.

In the proposed adaptive EKF, magnetic disturbances are estimated as a random vector and be tuned by modifying the parameters of the time constant matrix. It can be observed from Figure 3 and Figure 4, regarding the Bland–Altman plot between the reference and the adaptive EKF, that the fine alignment first starts from the Euler angles derived from coarse alignment. After a few iterations of filtering, the heading angles convergence is quick; however, it varies on the scenarios.

In Table 2, compared with the heading results derived from coarse alignment shown in Table 1, the heading angles converge to the reference in all the scenarios after the fin alignment process. This demonstrates the effectiveness of the fine alignment EKF in estimating and compensating the magnetic disturbances. The accuracy of the headings is reached at an average error of 2.9°. Regarding the pitch and roll angles, the pitch angle presents an average error of 0.5° compared with the references; the roll angles have an average error of 0.5°. In conclusion, the proposed adaptive EKF presents a significant improvement in the heading attitude by compensating magnetic disturbances.

### 4.2. AHRS Estimation in a Long-Term Test

The adaptive EKF used by the fine alignment process is also employed in the attitude estimation. After the completion of a fine alignment, the adaptive EKF automatically works in the dynamic estimation of attitude.

**Figure 5:**
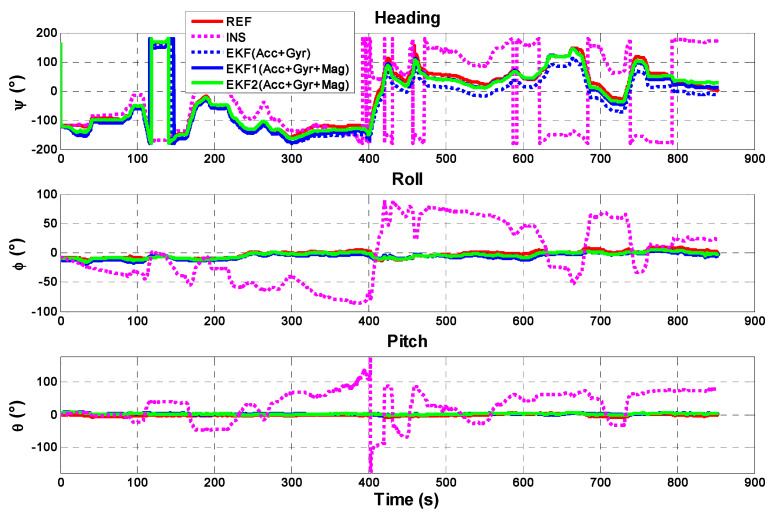
The attitude angles derived from different attitude estimation solutions.

Tests are performed to validate the performance of the proposed EKF under a dynamic scenario. The attitude in this study is determined by three methods: (1) dead-reckoning, i.e., the angular velocity from gyroscopes is integrated into standalone mode, namely INS; (2) attitude iterative estimation by EKF that considers only accelerometers and gyroscopes for integration, namely EKF(Acc+Gyr), (3) attitude iterative estimation by EKF that considers accelerometers, gyroscopes and magnetometers for integration without coarse and fine alignments, namely EKF1(Acc+Gyr+Mag), (4) attitude iterative estimation by EKF that considers accelerometers, gyroscopes and magnetometers for integration with initialization coarse and fine alignments, namely EKF2(Acc+Gyr+Mag) discussed in Section 3; (5) Vicon optical system used as the reference. The attitude results represented by Euler angles are given in Figure 5.

As can be seen in the figure, the drift in the attitude solution derived from INS without considering any additional method for calculation is significant. The heading, pitch and roll angles rapidly diverge from the reference. This figure also presents that while EKF(Acc+Gyr), EKF1(Acc+Gyr+Mag), and EKF2 (Acc+Gyr+Mag) outperform INS solution due to their partial usage of IMU data or initialization methods (coarse and fine alignments), they cannot reach the reference performance.

**Figure 6:**
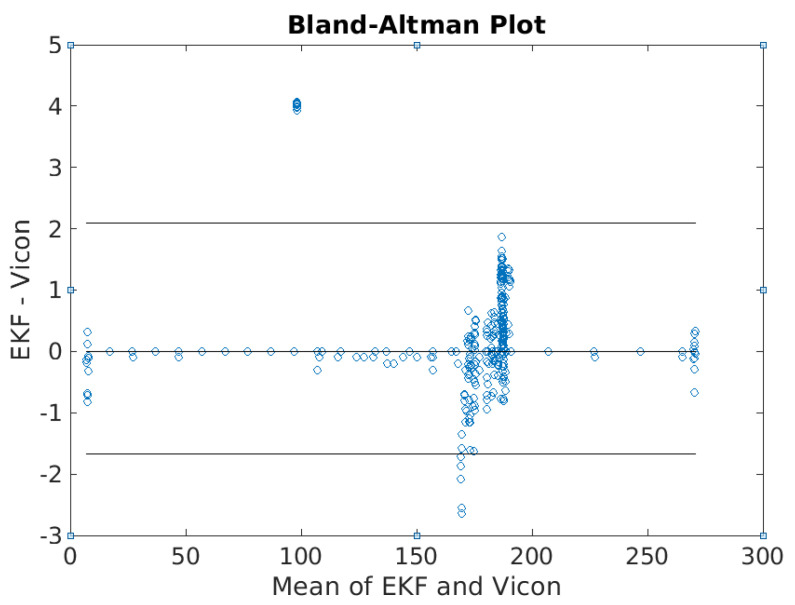
Bland–Altman plot of heading results ψ (⸰) derived from attitude EKF2.

**Figure 7:**
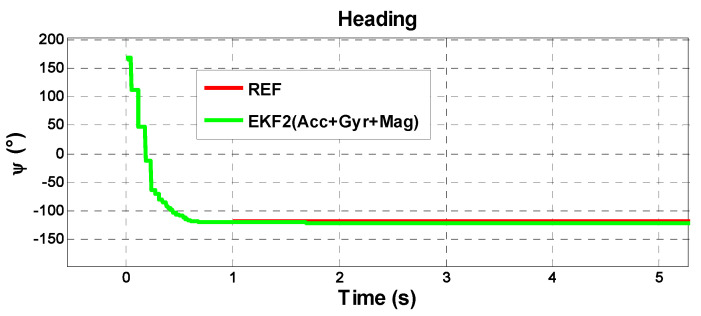
The initial transients of heading estimates in EKF2.

Compared with the previous solutions, the attitude EKF2 presents a good estimation of the attitude. Figure 6, which considers the first 300 s of the test, shows that the heading angles converge to the heading reference at 187° after the fine alignment and the transition from fine alignment to the attitude estimation process is automatic. The initial transients generated from the fine alignment are depicted in Figure 7, where it is seen that the heading is converged in less than one second. The average error in the heading angles is 2.5° compared with the reference. One can also realize that as the iteration proceeds, the attitude errors are reduced gradually. For example, the average error during the time 0~150 s is less than 1.0°; whereas it is 2.0° during 140~270 s.

The max error (6°) in the heading is caused by the errors in the gravity measurements used in Kalman filtering and the cross-axis sensitivity of the inertial sensors. A small error in the gravity measurements has a significant impact on the heading. The errors in the heading are coupled into pitch and roll because of the sensor cross-axis sensitivity. This increases the errors in the gravity measurements due to the deteriorated pitch and roll solutions.

**Figure 8:**
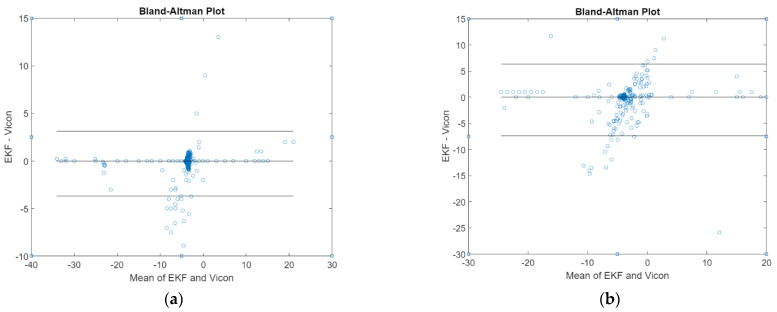
Bland–Altman plot of (**a**) pitch θ (⸰) angle, and (**b**) roll ϕ (⸰) angle derived from attitude EKF2.

The Bland–Altman plot of pitch and roll angles obtained from the attitude EKF2 presented in Figure 8 shows the average errors of 0.7° in both. Compared with the fine alignment process, the attitude EKF2 reaches the same accuracy in estimating pitch and roll under dynamic conditions. The errors caused by the rotations introduced during the testing are not exactly about the sensor axes; as a result, the rotation along one axis is projected into the other two axes. Moreover, the EKF2-derived attitude is noisier compared to the reference because the EKF2 is tuned to have a wide bandwidth and thus a quick dynamic response.

In conclusion, the attitude EKF2 presents a stable and accurate estimation of the attitude referring to the reference. This demonstrates the feasibility of applying such an EKF2 in estimating attitude when using the low-cost inertial sensors.

## 5. Conclusions

This paper developed and validated an AHRS estimation algorithm in navigation and tracking systems using low-cost and small-scale MEMS-based inertial sensors. The attitude estimation process incorporates the mechanism for adapting the EKF in the presence of sensor acceleration and magnetic disturbances. In the experiment, improvement in the accuracy of the initial orientation as significant as 45% is obtained from the new method as compared with other techniques. While the performance of this method is superior to the other techniques, the errors in the derived initial orientation are sometimes as much as 8°. These errors affect the subsequent attitude estimation process and may result in the deterioration of the attitude estimates. So, future work will investigate the impact of the initial errors on the attitude estimates. This will help indicate the sources of the initial errors and interpret the errors in the attitude estimation process, which in turn will improve the accuracy of the estimated orientation.

## Figures and Tables

**Table 1:** Mean error in coarse alignment process.

#	References			Geomagnetic Matching			Compass Heading			Hybrid Method		
	ϕ	θ	ψ	ϕ	θ	ψ	ϕ	θ	ψ	ϕ	θ	ψ
1	−1.4	−1.2	91.3	−4.7	−0.7	257.1	−1.5	−0.9	257.0	−1.5	−0.9	257.1
2	−1.9	0.9	24.1	−0.6	1.0	200.2	−1.7	−0.3	200.2	−1.7	−0.3	200.2
3	−1.9	−0.4	176.4	−0.7	6.3	357.8	−2.4	−1.0	357.8	−2.4	−1.0	357.8
4	−2.1	21.0	106.8	−7.7	21.7	268.7	−2.8	20.7	268.7	−2.8	20.7	268.7
5	0.6	−8.6	71.2	1.4	−9.0	242.3	1.4	−9.0	242.3	1.4	−9.0	242.3
6	1.2	32.9	194.4	2.5	39.0	14.0	−0.7	34.5	14.0	−0.7	34.5	14.02
7	5.0	−5.6	169.2	6.7	0.6	357.2	5.4	−5.5	357.2	5.4	−5.5	357.2
8	−10.1	−4.9	161.9	−10.1	2.1	345.4	−10.1	−4.6	345.4	−10.1	−4.6	345.4

**Table 2:** Mean error in fine alignment process.

Scenario	References			Estimated		
	ϕ	θ	ψ	ϕ	θ	ψ
1	−1.4	−1.2	91.3	−1.6	−0.8	90.8
2	−1.9	0.9	24.1	−1.8	−0.4	27.6
3	−1.9	−0.4	176.4	−2.4	−0.9	174.5
4	−2.1	21.0	106.8	−3.0	20.7	102.6
5	0.6	−8.6	71.2	1.1	−9.0	72.0
6	1.2	32.9	194.4	−0.7	34.5	191.4
7	5.0	−5.6	169.2	5.3	−5.4	173.6
8	−10.1	−4.9	161.9	−10.3	−4.5	164.6

## Data Availability

Data sharing is not applicable to this article.
